# New Ethynylphenylborasilsesquioxanes—Their Reactivity and Behavior during Thermal Decomposition

**DOI:** 10.3390/ijms241813960

**Published:** 2023-09-11

**Authors:** Miłosz Frydrych, Bogna Sztorch, Robert E. Przekop, Bogdan Marciniec

**Affiliations:** 1 Faculty of Chemistry, Adam Mickiewicz University, 8 Uniwersytetu Poznańskiego, 61-614 Poznań, Polandmarcinb@amu.edu.pl (B.M.); 2 Centre for Advanced Technologies, Adam Mickiewicz University, 10 Uniwersytetu Poznańskiego, 61-614 Poznań, Poland; bogna.sztorch@amu.edu.pl

**Keywords:** POSS, borasilsesquioxanes, heterosilsesquioxanes, hydrosilylation, thermal decomposition, thermal analysis

## Abstract

In this paper, a new type of borasilsesquioxanes was synthesized through a condensation process, and its reactivity in catalytic hydrosilylation reactions with silanes, siloxanes, and silsesquioxanes was investigated. The obtained compounds were mostly obtained in >90% yield. They were fully characterized using spectroscopic (^1^H, ^13^C, ^29^Si NMR) and spectrometric (MALDI-TOF-MS) methods. The next stage of the research involved studying the thermogravimetric properties of the borasilsesquioxanes. By analyzing the different stages of decomposition using spectroscopic techniques (NMR, ATR-FTIR, Raman) and microscopic imaging, it was found that the structure of the borasilsesquioxanes changed during the pyrolysis process and polymer compounds were formed.

## 1. Introduction

Silsesquioxanes are hybrid organosilicon compounds with the general formula [RSiO_1.5_]_n_, where *R* is the arbitrary organic group linked to a silicon atom. Their unique cube-like structure creates a three-dimensional space, consisting of an inorganic core and attached organic functional groups [1,2,3,4]. Modifying the functional groups of these compounds allows one to adjust the product’s properties by synthesis [5,6,7]. Typically, functionalization occurs through catalytic reactions like hydrosilylation, silylative coupling, and metathesis [8,9,10,11,12]. As a result, these compounds have received significant attention in the field of materials chemistry and nanotechnology [13,14,15]. They found many applications, including composite materials (as an additive that considerably improves thermal and mechanical properties), catalysis, biomedical applications (drug carriers, dentistry), surface coatings, optoelectronics, microelectronics, etc. [16,17,18,19,20,21,22,23,24,25,26,27,28].

A special group of silsesquioxanes is the so-called heterosilsesquioxanes. The compound has one or more heteroatoms in its cage structure or corner, replacing the silicon atom. This results in some new properties for the compound. The first literature reports on structures of this type include the work of Fehrer et al., who obtained heterosilsesquioxanes containing germanium, tin, and zirconium in their research using the corner capping reaction from trisilanol, as analogs of transition metal catalysts supported on silica [29,30]. Other examples of metallasilsesquioxanes known in the literature are compounds containing zinc, aluminum, vanadium, and hafnium [31,32,33].

In this work, heterosilsesquioxanes containing a boron atom in their structure will be discussed—borasilsesquioxanes. The first work focusing on obtaining such compounds was performed by Fehrer et al. and Duchateu et al., who received a borasilsesquioxanes dimer [34,35]. An important study was conducted by Maleczka et al., in which they successfully synthesized asymmetric double-deckers containing borasilsesquioxanes through a condensation reaction [36].

In previous papers [37,38], we described the methods of obtaining and functionalizing mono- and distyrylborasilsesquioxane by catalytic reactions, i.e., hydrosilylation and metathesis. Entirely new structures were obtained, which were confirmed and characterized spectroscopically. In addition, it has been proven that the products obtained undergo a spontaneous redistribution reaction of the alkoxide group from silane to borane. The high conversions and ease of modification prompted us to obtain new derivatives containing a triple bond, which, as we predicted, is characterized by a much higher reactivity. In this work, we synthesized mono- and diethynylphenylborasilsesquioxane by the condensation reaction. Furthermore, this is a continuation of our team’s previous work on thermally inducted chemical and structural transformations of silsesquioxanes and heterosilsesquioxanes [7,39,40,41,42].

The objective of this study was to synthesize borasilsesquioxane derivatives through a process of catalytic hydrosilylation, and subsequently analyze and identify the properties of these synthesized compounds. The new compounds were fully characterized using spectroscopic and spectrometric analysis (^1^H, ^13^C, ^29^Si NMR, and MALDI-TOF-MS). Thermogravimetric studies were also conducted to analyze the thermal decomposition of this class of compounds and evaluate their potential application.

## 2. Results and Discussion

### 2.1. Reactivity Tests

**Figure 1:**
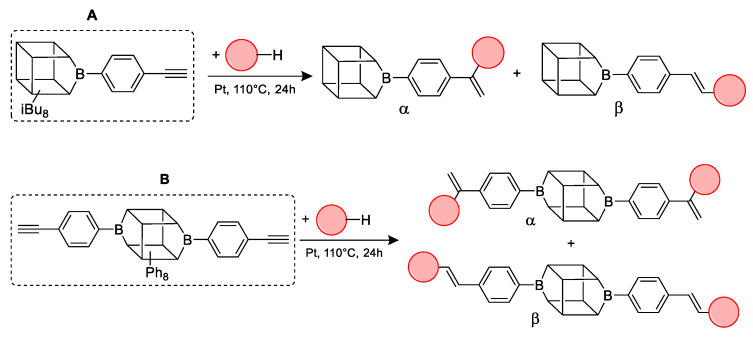
General hydrosilylation scheme of (**A**) monoethynylphenylborasilsesquioxane and (**B**) diethynylphenylborasilsesquioxane with compound bearing Si–H moiety.

In the presented work, 4-ethynylphenylsilsesquioxanes were obtained by condensation with silanols, similarly to in our previous articles [37,38]. The next step involved functionalization by the catalytic hydrosilylation of the ethynyl bond (Figure 1). Several ethynylphenylsilsesquioxane derivatives were synthesized and analyzed using spectroscopic techniques (NMR, MALDI-TOF-MS). This document presents the first attempt at the preparation and functionalization this type of compound.

Substrates containing the Si–H bond were selected to contain a full cross-section of increasingly complex organosilicon compounds: silanes, siloxanes, and silsesquioxanes. Compounds A and B were purified prior to the catalytic tests. Compound A was derived by freezing in fresh liquid nitrogen followed by grinding and drying on a Schlenk line. Compound B was derived by dissolving unreacted condensation reactants in boiling acetonitrile, hot filtration, and then drying the product on a Schlenk line.

Catalytic tests were carried out using a Karstedt catalyst. This is a stable, readily available, and highly active catalyst that is widely used in many modern applications [43].

In the first stage of catalytic tests, the reactivity of A with simple compounds containing the Si–H bond—triethyl and triethoxysilane—was investigated (Table 1). Compared to the vinyl equivalent of silsesquioxane, the reaction with trimethylsilane occurs, and this may be due to the higher polarity of the ethynyl group and the strong electron-withdrawing effect [44]. In the case of a reaction with triethoxysilane, a complex reaction mixture is also formed and a back-biting reaction takes place, i.e., a redistribution reaction involving the transfer of an alkoxy group from silane to boron, occurring between siloxyboranes and alkoxysilanes [38]. The high conversion in reactions with siloxanes (PDMS, HMTS, TMDS-OD) prompted us to perform tests with more complex structures—silsesquioxanes. In the case of the reaction with octaspherosilicate, mainly the β product was obtained, which is caused by steric hindrance in the formation of the α product.

Table 2 summarizes the reactions for compound B, indicating its higher reactivity compared to compound A. All reactions resulted in >90% conversion even at an equimolar ratio. Similar to compound A, back-biting was observed in the reaction with triethoxysilane, leading to the formation of a complex reaction mixture.

### 2.2. Thermal Decomposition Analysis

**Figure 2:**
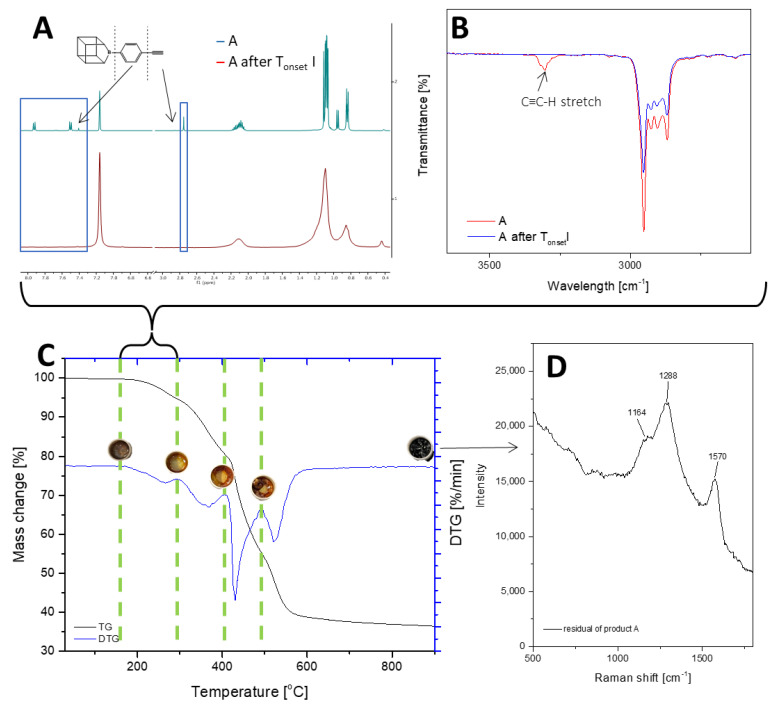
TGA and DTG curves of A—monoethynylphenylborasilsesquioxane in a nitrogen atmosphere (**C**). The graph has been divided into stages of decomposition. Additional photos and spectroscopy analysis (^1^H NMR (**A**), FT-IR (**B**), Raman (**D**)) show the sample’s appearance and changes in structure before individual stages.

**Figure 3:**
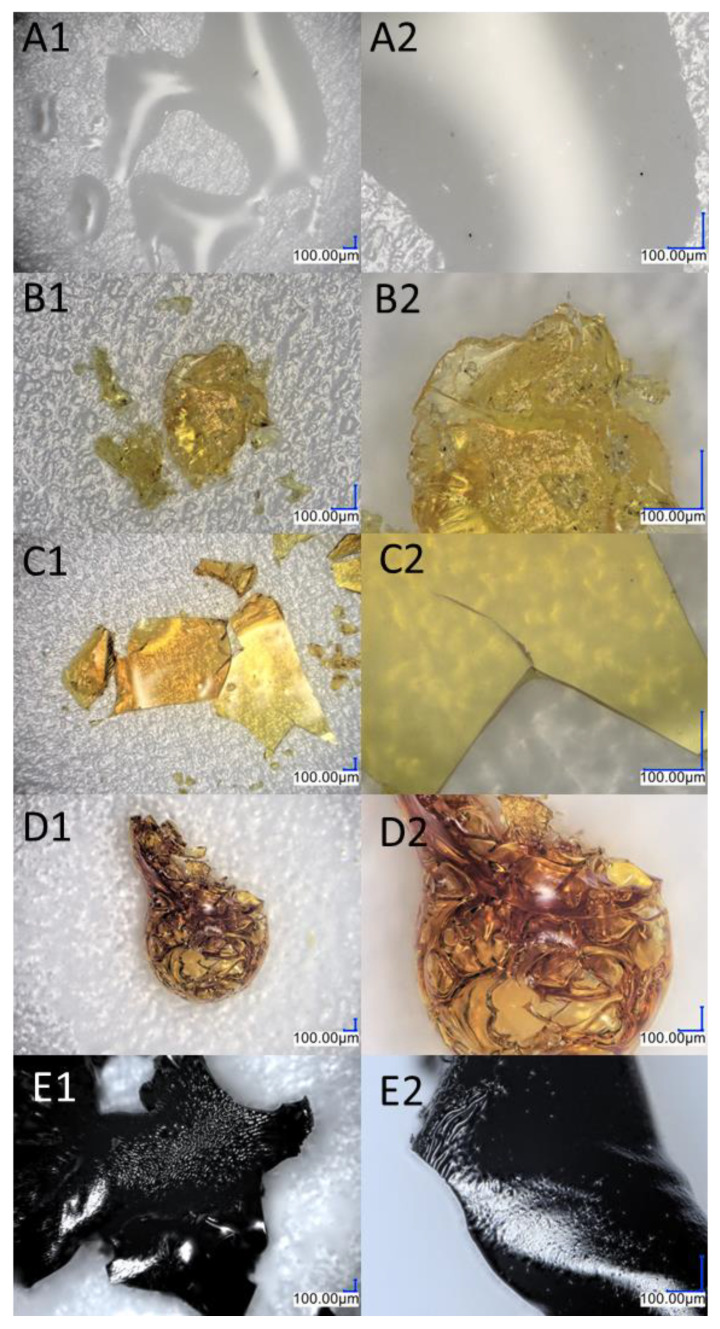
Images from an optical microscope depicting the various stages of the monoethynylphenylborasilsesquioxane decomposition process. (**A1**,**A2**) Before first stage of degradation; (**B1**,**B2**) after first stage of degradation; (**C1**,**C2**) after the second stage of degradation; (**D1**,**D2**) after the third stage of degradation; (**E1**,**E2**) residual after the process.

**Figure 4:**
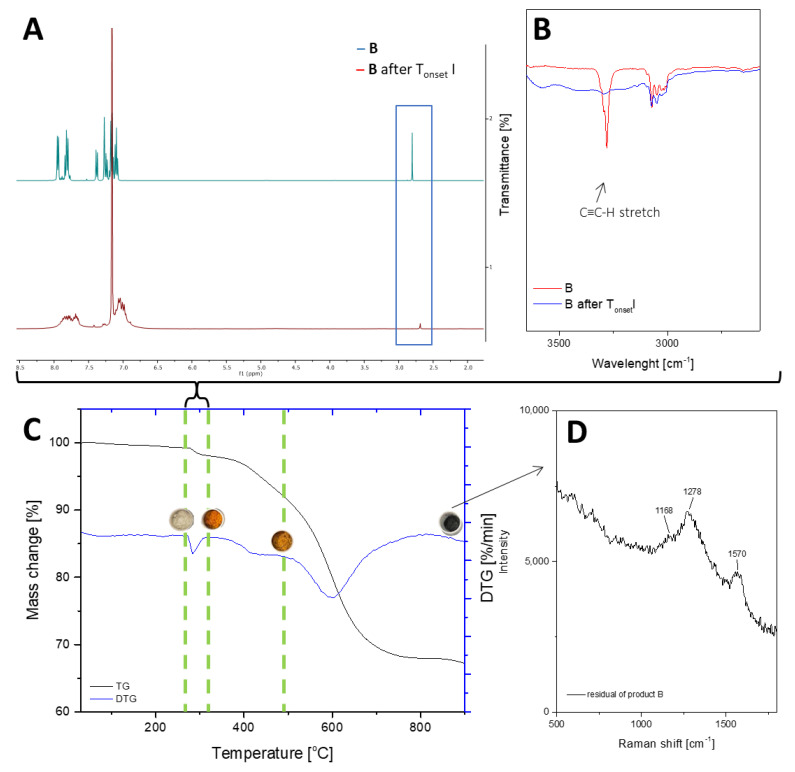
TGA and DTG curves of B—diethynylphenylborasilsesquioxane in a nitrogen atmosphere (**C**). The graph has been divided into stages of decomposition. Additional photos and spectroscopy analysis (^1^H NMR (**A**), FT-IR (**B**), Raman (**D**)) show the sample’s appearance and changes in structure before individual stages.

**Figure 5:**
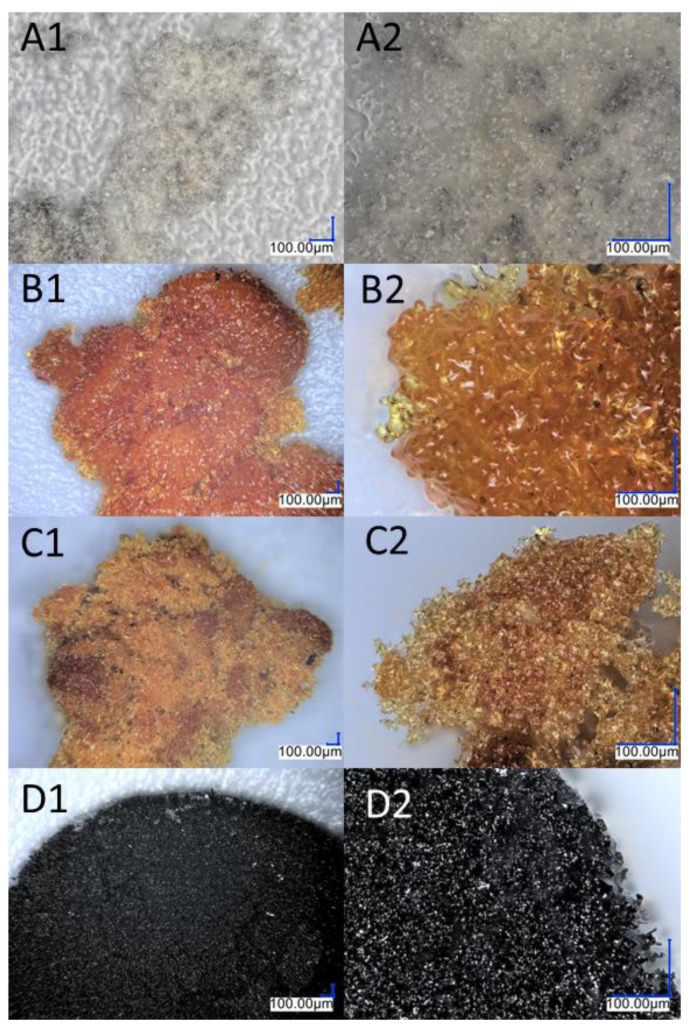
Images from an optical microscope showing the various stages of the diethynylphenylborasilsesquioxane decomposition process. (**A1**,**A2**) Before the first stage of degradation; (**B1**,**B2**) after the first stage of degradation; (**C1**,**C2**) after the second stage of degradation; (**D1**,**D2**) residual after the process.

On the basis of the obtained thermogravimetric measurements in an inert atmosphere (N_2_), microscopic photos of the samples were taken after the individual stages of decomposition, and the curves (Figure 2, Figure 3, Figure 4 and Figure 5) and the parameters T_1%_, T_onset_ and T_max_ for compounds A and B were determined (Table 3).

The thermogravimetric measurement indicates that the decomposition of compounds A and B occurs in four stages. T_1%_ signifies that the samples are free from impurities and can maintain stability until the first stage of degradation. Sample A is a solid with a waxy texture that transforms into a fluid, viscous, and colorless liquid when heated to approximately 180 °C (Figure 3A1,A2). The melting point of compound A was determined as 43.6 °C. The thermal decomposition is related to the cracking of bonds. As we proved in the previous work, decomposition at lower temperatures occurs in the vicinity of the silsesquioxane core (usually the attached functional group). As the temperature rises, the fragmentation of the core of the cube may occur. However, it should be noted that in the case of such complex systems, these effects can overlap and flow smoothly. The first stage of decomposition A starts at 224.2 °C, then most likely the decomposition of isobutyl groups begins, and this ends after the third stage of decomposition to 512.4 °C. In addition, there is a change in the color of the sample, which is associated with the decomposition of the ethynylphenyl group and was confirmed using spectroscopic methods. The FT-IR spectra indicate that the ≡C–H stretching vibrations responsible for the band at 3300 cm^−1^ have disappeared, as verified by NMR spectroscopy, which also confirmed the disappearance of the triple and phenyl bonds (see Figure 2). Then, the core of the cube was fragmented, and after the pyrolysis process, silica and coke remained. Raman spectroscopy was performed to verify the residual products after the process, with bands around 1160 cm^−1^ indicating the Si–O bond from the cube core [45]. The other two bands are attributed to the stretching vibration of sp^2^ carbon atoms [46,47]. The microscopic analysis demonstrates how the sample changes from liquid to solid, with a change in color (Figure 3B1,B2), and then transforms into a glass-like structure (Figure 3C1–D2). The final pyrolysis product is a black, brittle solid (Figure 3E1,E2).

**Figure 6:**
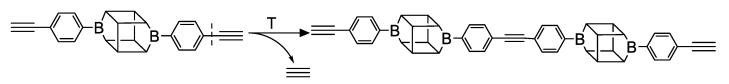
Scheme for the formation of polymer structures during the thermal decomposition of diethynylphenylborasilsesquioxane.

As previously mentioned, Sample B also underwent four stages of thermal decomposition; however, the second and third stages overlapped. There are some noticeable differences between Sample B and Sample A, such as the decomposition start temperature and the residual mass of the compound. This is due to the fact that silsesquioxanes with phenyl groups are among the most thermally stable compounds of their class due to the high content of aromatic groups [48]. Moreover, the increased residue content may be a result of carbon being trapped in the structure, which could be due to the condensation of the phenyl group [49]. The first stage of decomposition began at 278 °C, which is related to the melting point, 279.7 °C. During this slight mass change, which corresponds to the removal of acetylene from the structure, the sample changed color (Figure 5A1–B2). FT-IR studies before and after the process, as well as the change in the weight of the sample, indicate that there was a partial disappearance of the HC≡C triple bonds, which can also be seen in the NMR spectra. The resulting product (Figure 5B1,B2) was a partially soluble compound that formed a suspension in solvents. The NMR spectra do not indicate the formation of double bond signals. The above-mentioned changes that occurred in the spectroscopy tests as well as the mass loss corresponding to the outgoing acetylene indicate the formation of polymeric structures. The incomplete disappearance of the band in the spectra suggests the formation of the product shown in Figure 6. The subsequent overlapping stages are responsible for breaking the bonds between the core and phenyl groups, followed by the fragmentation of the cage itself. The pyrolysis product containing silica and coke is a black compacted material that resembles a glass-like structure when magnified (Figure 5D1,D2). Raman spectroscopic analysis showed analogous results as were seen in the case of product A.

## 3. Materials and Methods

### 3.1. Materials

The chemicals were purchased from the following sources:

Disilanolisobuthyl POSS and Tetrasilanolphenyl POSS from Hybrid Plastic, Hattiesburg, MI, USA; 4-ethynylphenylboronic acid (95%), platinum catalyst (Pt_2_(dvds)_3_), tetramethoxydisiloxane (97%), Triethylsilane Et_3_SiH (99%), Triethoxysilane (EtO)_3_SiH (95%), Pentamethylsiloxane PMDS (95%), Heptamethylsiloxane HMTS (97%) and Dimethylphenylsilane Me_2_PhSiH (98%) from Merck Millipore, Darmstadt, Germany; toluene from Chempur, Piekary Slaskie, Poland; benzene-d6 from Deutero, Gdansk, Poland. Toluene was dried over P_2_O_5_, distilled under argon and stored under argon atmosphere in Rotaflo Schlenk flasks over Na/K alloy.

### 3.2. Synthesis of Organosilicon Precursors

Monospherosilicate SSQ-OSiH was synthesized according to the procedure given in the literature [50] with an isolated yield of 91% based on heptaisobutyltrisilanol.

Octaspherosilicate SS-8H was synthesized according to the procedure given in the literature [51]. The product was obtained with a 95% yield.

TMDS-OD (1,3,3-tetramethyloctadecyldisiloxane)—100 g TMDS was dissolved in 500 mL hexene, the system was brought to a boil, then octadecene containing Karstedt’s catalyst (10–5 Pt/per mol Si-H) was added continuously while keeping the boiling point. The reactions were then sampled via NMR spectroscopy until complete olefin conversion—2 h (TMDS 5eq: octadecene 1eq)—was observed. The reactions were evaporated to a pure product residue.

Monosubstituted and diethynylphenyllborasilsesquioxane were obtained by a condensation reaction of Disilanolisobuthyl POSS and Tetrasilanolphenyl POSS, respectively, with 4-ethynylphenylboronic acid, with the azeotrophic elimination of stoichiometrically formed water. This preparation strategy enables the obtaining of quantitative yields, with the details described in our previous work [37,38].

#### 3.2.1. General Procedure for Hydrosilylation of Ethynylphenylborasilsesquioxane with Compounds Bearing Si–H Moiety

All hydrosilylation reactions were conducted under an argon atmosphere in 25 mL high-pressure Schlenk reactors equipped with a Rotaflo stopcock and magnetic stirring bars. In a typical procedure, a Schlenk’s reactor was charged with 0.056 mmol (50 mg) of ethynylphenylborasilsesquioxane, 3 mL of toluene and an equimolar quantity of a given compounds bearing Si–H moiety (in accordance to tests summarized in Table 1). Karstedt’s catalyst solution (10^−5^ eq Pt/mol Si-H) was added. The reaction mixture was set at 110 °C for 24 h. After the removal of the solvent under reduced pressure, ^1^H NMR analysis was run to measure conversion rate and product selectivity.

#### 3.2.2. General Procedure for Hydrosilylation of Diethynylphenylborasilsesquioxane with Compounds Bearing Si–H Moiety

All hydrosilylation reactions were conducted under argon atmosphere in 25 mL high-pressure Schlenk reactors equipped with a Rotaflo stopcock and magnetic stirring bars. In a typical procedure, a Schlenk’s reactor was charged with 0.056 mmol (50 mg) of diethynylphenylborasilsesquioxane, 3 mL of toluene and equimolar quantity of a given compounds bearing Si–H moiety (in accordance with tests summarized in Table 1). Karstedt’s catalyst solution (10^−5^ eq Pt/mol Si-H) was added. The reaction mixture was set at 110 °C for 24 h. After the removal of the solvent under reduced pressure, the ^1^H NMR analysis was run to measure the conversion rate and product selectivity.

### 3.3. NMR Spectroscopy Analysis


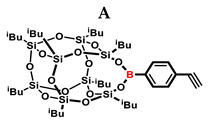


**^1^H NMR** (400 MHz, C_6_D_6_): δ (ppm) = 7.93 (d, J = 8.1 Hz, 2H, B-C_6_H_4_), 7.50 (d, J = 8.1 Hz, 2H, B-C_6_H_4_), 2.75 (s, 1H, C≡C-H) 2.22–2.02 (m, 8H, -CH_2_-CH(CH_3_)_2_), 1.12–1.07 (m, 48H, -CH_2_-CH(CH_3_)_2_), 0.95 (d, J = 7.1 Hz, 4H, -CH_2_-CH(CH_3_)_2_, 9 and 13 position isobutyl substituents), 0.85–0.83 (m, 12H, -CH_2_-CH(CH_3_)_2_).

**^13^C NMR** (101 MHz, CDCl_3_): δ (ppm) = 135.60, 131.70, 128.41, 125.58, (Ph), 84.04, 79.11 (C≡C) 26.12, 26.06, 25.98, 25.94, 24.66, 24.49, 24.45, 24.25, 23.49, 23.03 (iBu).

**^29^Si NMR** (79.5 MHz C_6_D_6_): δ (ppm) = −66.82 (9 and 13 position), −67.90 (1, 3, 7 and 15 position), −69.92 (5 and 17 position).

**MALDI-TOF-MS:** [M] + Na^+^: 1023.342 (measured), 1023.350 (calculated).


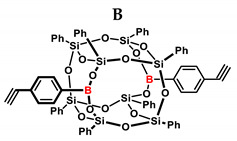


**^1^H NMR** (400 MHz, C_6_D_6_): δ (ppm) = 7.95–7.93 (m, 8H, B-C_6_H_4_-), 7.84–7.79 (m, 12H, Ph) 7.40–7.07 (m, 20H, Ph, solvent), 2.80 (s, 2H, C≡C-H).

**^13^C NMR** (101 MHz, CDCl_3_): δ (ppm) = 136.14, 134.56, 134.53, 134.51, 134.48, 131.61, 131.50, 131.18, 131.10, 130.63, 128.49, 127.82, 125.77 (Ph), 84.00, 79.18 (C≡C).

**^29^Si NMR** (79.5 MHz C_6_D_6_): δ (ppm) = −77.70, −79.45 (cage).

**MALDI-TOF-MS:** [M] + Na^+^: 1288.160 (measured), 1288.154 (calculated).


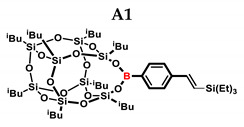


**^1^H NMR** (400 MHz, C_6_D_6_): δ (ppm) = 8.12–8.07 (m, 2H B-C_6_H_4_) 7.43–7.41 (d), 7.31–7.29 (d) (2H, B-C_6_H_4_) 7.02–6.97 (d, J = 19.47 Hz, β isomer, B-C_6_H_4_-CH_=_CH-Si), 6.54–6.49 (d, J = 19.26 Hz, β isomer, B-C_6_H_4_-CH_=_CH-Si) 5.88 (d, J = 3.11 Hz, α isomer B-C_6_H_4_-C(_=_CH_2_)-Si), 5.54–5.53 (d, J = 3.14 Hz, α isomer, B-C_6_H_4_-C(_=_CH_2_)-Si), 2.26–2.15 (m, 2H, -CH_2_-CH(CH_3_)_2_, 9 and 13 position isobutyl substituents, α isomer B-C_6_H_4_-CH(CH_3_)-Si), 2.15–2.03 (m, 6H, -CH_2_-CH(CH_3_)_2_), 1.16–1.12 (m, 12H, -CH_2_-CH(CH_3_)_2_, 9 and 13 position isobutyl substituents), 1.10–1.06 (m, 36H, -CH_2_-CH(CH_3_)_2_),1.03–0.90 (m, 16H, -CH_2_-CH(CH_3_)_2_), 0.86–0.83 (m, 9H, Si-CH_2_-CH_3_), 0.68–0.62 (q, 6H, Si-CH_2_-CH_3_).

**^13^C NMR** (101 MHz, C_6_D_6_): δ (ppm) = 150.64, 148.69, 145.36, 141.16, 135.79, 135.60, 128.90, 126.81, 126.12, 125.83, 125.30(Ar), 25.80, 25.78, 25.69, 25.60, 25.56, 25.53, 24.33, 24.10, 24.07, 24.00, 23.97, 23.12, 22.66 (iBu), 7.31(CH_3_), 3.51(CH_2_).

**^29^Si NMR** (79.5 MHz, C_6_D_6_): δ (ppm) = 2.33, −0.16 (SiEt_3_), −66.83, −67.94, −69.97 (cage)

**MALDI-TOF-MS:** [M] + Na^+^: 1139.455 (measured), 1139.452 (calculated).


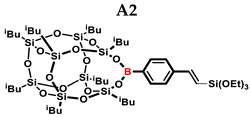


**^1^H NMR** (400 MHz, C_6_D_6_): δ (ppm) = 8.12–7.39 (m, 4H B-C_6_H_4_) 7.02–6.97 (d, J = 19.47 Hz, β isomer, B-C_6_H_4_-CH_=_CH-Si), 6.59–6.54 (d, J = 19.22 Hz, β isomer, B-C_6_H_4_-CH_=_CH-Si) 6.18 (d, J = 3.11 Hz, α isomer B-C_6_H_4_-C(_=_CH_2_)-Si), 6.11 (d, J = 3.14 Hz, α isomer, B-C_6_H_4_-C(_=_CH_2_)-Si), 4.30–3.70 (m, OCH_2_CH_3_), 2.22–2.02 (m, 8H, -CH_2_-CH(CH_3_)_2_), 1.16–1.04 (m, -CH_2_-CH(CH_3_)_2_), 0.85–0.82 (m, CH_2_-CH(CH_3_)_2_).

**^13^C NMR** (101 MHz, C_6_D_6_): δ (ppm) = 135.76, 135.64, 135.20, 133.37, 131.29, 126.68, 126.08, 125.18 (Ar), 29.83, 25.83, 25.73, 25.71, 25.56, 24.26, 24.12, 23.85, 23.31, 23.10, 22.63, 18.05 (iBu).

**^29^Si NMR** (79.5 MHz, C_6_D_6_): δ (ppm) = −66.59, −66.63, −66.84, −66.88 (SiOEt), −67.92, −67.99, −68.43, −68.49, −68.61, −68.76, −69.94, −70.18 (cage), −72.83, −76.00 (SiOEt).

**MALDI-TOF-MS:** [M] + Na^+^: 1187.440 (measured), 1187.437 (calculated).


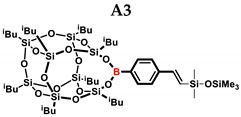


**^1^H NMR** (400 MHz, C_6_D_6_): δ (ppm) = 8.13–8.05 (m, B-C_6_H_4_) 7.50–7.41 (m, B-C_6_H_4_) 7.09–7.04 (d, J = 19.08 Hz, β isomer, B-C_6_H_4_-CH_=_CH-Si), 6.57–6.52 (d, J = 19.23 Hz, β isomer, B-C_6_H_4_-CH_=_CH-Si) 5.90–5.89 (d, J = 2.77 Hz, α isomer B-C_6_H_4_-C(_=_CH_2_)-Si), 5.68–5.67 (d, J = 2.76 Hz, α isomer, B-C_6_H_4_-C(_=_CH_2_)-Si), 2.24–2.15 (m, 2H, -CH_2_-CH(CH_3_)_2_, 9 and 13 position isobutyl substituents, α isomer B-C_6_H_4_-CH(CH_3_)-Si), 2.12–2.05 (m, 6H, -CH_2_-CH(CH_3_)_2_), 1.15–1.13 (d, 12H, -CH_2_-CH(CH_3_)_2_, 9 and 13 position isobutyl substituents), 1.10–1.06 (m, 36H, -CH_2_-CH(CH_3_)_2_),1.02–0.99 (m, 4H, -CH_2_-CH(CH_3_)_2_, 9 and 13 position isobutyl substituents), 0.85–0.80 (m, 12H, -CH_2_-CH(CH_3_)_2_), 0.26 (s, β isomer SiMe_2_), 0.24 (s, α isomer SiMe_2_), 0.15 (s, β isomer SiMe_3_), 0.07 (s, α isomer SiMe_3_).

**^13^C NMR** (101 MHz, C_6_D_6_): δ (ppm) = 153.47, 147.45, 145.22, 141.52, 136.45, 136.23, 135.83, 131.93, 130.23, 129.57, 126.62, 125.82 (Ar), 26.44, 26.43, 26.38, 26.34, 26.32, 26.25, 26.23, 26.20, 24.96, 24.90, 24.74, 24.71, 24.62, 24.60, 24.49, 23.76, 23.73, 23.30, 23.27 (iBu), 2.43, 2.23, 1.45, 1.24. (SiMe_2_, SiMe_3_).

**^29^Si NMR** (79.5 MHz, C_6_D_6_): δ (ppm) = 8.41, 8.18 (OSiMe_3_), −3.15, −3.56 (OSiMe_2_), −66.85, −67.98, −68.01, −70.02, −70.07 (cage).

**MALDI-TOF-MS:** [M] + Na^+^ + H•: 1172.447 (measured), 1172.432(calculated).


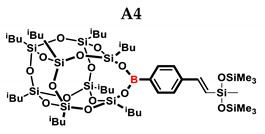


**^1^H NMR** (400 MHz, C_6_D_6_): δ (ppm) = 8.14–8.12 (m, α isomer, B-C_6_H_4_), 8.06–8.04 (m, β isomer, B-C_6_H_4_) 7.58–7.56 (α isomer, B-C_6_H_4_) 7.44–7.42 (β isomer, B-C_6_H_4_), 6.52–6.47 (d, J = 19.22 Hz, β isomer, B-C_6_H_4_-CH_=_CH-Si), 5.96–5.95 (d, J = 2.56 Hz, α isomer, B-C_6_H_4_-C(_=_CH_2_)-Si), 5.80–5.79 (d, J = 2.33 Hz, α isomer, B-C_6_H_4_-CH_=_CH-Si), 2.25–2.16 (m, 2H, -CH_2_-CH(CH_3_)_2_, 9 and 13 position isobutyl substituents, α isomer B-C_6_H_4_-CH(CH_3_)-Si), 2.10–2.05 (m, 6H, -CH_2_-CH(CH_3_)_2_), 1.15–1.13 (d, 12H, -CH_2_-CH(CH_3_)_2_, 9 and 13 position isobutyl substituents), 1.10–1.06 (m, 36H, -CH_2_-CH(CH_3_)_2_),1.02–0.99 (m, 4H, -CH_2_-CH(CH_3_)_2_, 9 and 13 position isobutyl substituents), 0.85–0.80 (m, 12H, -CH_2_-CH(CH_3_)_2_), 0.30 (s, α isomer SiMe), 0.29 (s, β isomer SiMe), 0.20 (s, β isomer SiMe_3_), 0.11 (s, α isomer SiMe_3_).

**^13^C NMR** (101 MHz, C_6_D_6_): δ (ppm) = 151.36, 146.42, 145.87, 141.15, 136.24, 135.94, 135.60, 131.69, 128.71, 126.89, 126.40 (Ar), 30.24, 26.19, 26.10, 25.96, 24.70, 24.50, 24.47, 24.35, 23.52, 23.06 (iBu), 2.10, 1.91, 0.47, 0.34 (SiMe, SiMe_3_).

**^29^Si NMR** (79.5 MHz, C_6_D_6_): δ (ppm) = 8.26, 8.07 (OSiMe_3_), −33.37, −35.99 (OSiMe), −66.84, −67.93, −67.98, −69.92 (cage).

**MALDI-TOF-MS:** [M] + Na^+^ +: 1245.447 (measured), 1245.442 (calculated).


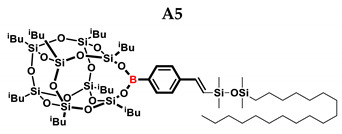


**^1^H NMR** (400 MHz, C_6_D_6_): δ (ppm) = 8.10–8.03 (m, B-C_6_H_4_) 7.48–7.42 (m, B-C_6_H_4_) 7.10–7.05 (d, J = 19.25 Hz, β isomer, B-C_6_H_4_-CH_=_CH-Si), 6.58–6.53 (d, J = 19.20 Hz, β isomer, B-C_6_H_4_-CH_=_CH-Si) 5.91–5.90 (d, J = 3.04 Hz, α isomer B-C_6_H_4_-C(_=_CH_2_)-Si), 5.70–5.69 (d, J = 3.05 Hz, α isomer, B-C_6_H_4_-C(_=_CH_2_)-Si), 2.22–2.12 (m, 2H, -CH_2_-CH(CH_3_)_2_, 9 and 13 position isobutyl substituents, α isomer B-C_6_H_4_-CH(CH_3_)-Si), 2.12–2.00 (m, 6H, -CH_2_-CH(CH_3_)_2_), 1.36–1.25 (m, 32H, octadecyl chain -CH_2_-) 1.15–1.13 (m, 12H, -CH_2_-CH(CH_3_)_2_, 9 and 13 position isobutyl substituents), 1.10–1.03 (m, 36H, -CH_2_-CH(CH_3_)_2_),1.00–0.98 (m, 4H, -CH_2_-CH(CH_3_)_2_, 9 and 13 position isobutyl substituents), 0.93–0.80 (m, 5H, octadecyl chain -CH_3_), 0.82–0.81 (m, 12H, -CH_2_-CH(CH_3_)_2_), 0.69–0.54 (m, 2H, octadecyl SiCH_2_-, SiCH-), 0.29–0.09 (m, SiMe_2_).

**^13^C NMR** (101 MHz, C_6_D_6_): δ (ppm) = 153.30, 147.21, 144.99, 141.27, 136.21, 136.00, 135.59, 131.68, 130.00, 128.30, 128.18, 128.06, 127.94, 127.82, 126.73, 126.36, 125.57 (Ar), 34.02, 33.98, 33.95, 32.40, 30.30, 30.27, 30.25, 30.19, 30.16, 29.99, 29.95, 29.92, 29.88, 26.22, 26.19, 26.15, 26.11, 26.09, 26.03, 26.00, 25.98, 24.72, 24.66, 24.50, 24.47, 24.38, 24.26, 23.90, 23.88, 23.78, 23.52, 23.49, 23.18, 23.06, 23.03 (iBu), 18.92, 18.88, 18.77, 14.44 (CH_2_,CH_3_), 1.30, 1.09, 0.76, 0.73, 0.53, 0.51 (SiMe_2_).

**^29^Si NMR** (79.5 MHz, C_6_D_6_): δ (ppm) = 8.80, 8.55, −3.38, −3.76 (OSiMe_2_), −66.88, −67.98, −67.99, −70.05 (cage).

**MALDI-TOF-MS:** [M] + Na^+^: 1409.692 (measured), 1409.690(calculated).


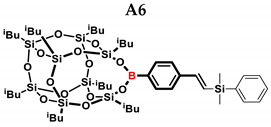


**^1^H NMR** (400 MHz, C_6_D_6_): δ (ppm) = 8.07–8.02 (m, 2H B-C_6_H_4_) 7.55–7.53 (m, 2H B-C_6_H_4_) 7.37–7.23 (d,d, 5H,Si-C_6_H_5_) 7.02–6.97 (d, J = 19.09 Hz, β isomer, B-C_6_H_4_-CH_=_CH-Si), 6.65–6.60 (d, J = 19.10 Hz, β isomer, B-C_6_H_4_-CH_=_CH-Si) 5.96–5.95 (d, J = 2.80 Hz, α isomer B-C_6_H_4_-C(_=_CH_2_)-Si), 5.62 (d, J = 2.84 Hz, α isomer, B-C_6_H_4_-C(_=_CH_2_)-Si), 2.24–2.16 (m, 2H, -CH_2_-CH(CH_3_)_2_, 9 and 13 position isobutyl substituents, α isomer B-C_6_H_4_-CH(CH_3_)-Si), 2.15–2.06 (m, 6H, -CH_2_-CH(CH_3_)_2_), 1.16–1.15 (d, 12H, -CH_2_-CH(CH_3_)_2_, 9 and 13 position isobutyl substituents), 1.10–1.06 (m, 36H, -CH_2_-CH(CH_3_)_2_),1.04–1.00 (m, 4H, -CH_2_-CH(CH_3_)_2_, 9 and 13 position isobutyl substituents), 0.87–0.80 (m, 12H, -CH_2_-CH(CH_3_)_2_), 0.36 (s, β isomer SiMe_2_), 0.34 (s, α isomer SiMe_2_).

**^13^C NMR** (101 MHz, C_6_D_6_): δ (ppm) = 151.72, 147.82, 145.96, 141.28, 138.60, 138.31, 136.16, 135.96, 134.60, 134.37, 129.65, 129.42, 128.67, 126.74, 126.40 (Ar), 26.20, 26.15, 26.09, 26.03, 26.00, 25.95, 24.72, 24.50, 24.47, 24.39, 23.52, 23.06 (iBu) −2.20, −2.40 (SiMe_2_).

**^29^Si NMR** (79.5 MHz, C_6_D_6_): δ (ppm) = 8.41, −10.57 (SiMe_2_), −66.82, −67.93, −69.96(cage).

**MALDI-TOF-MS:** [M] + Na^+^: 1159.423 (measured), 1159.421 (calculated).


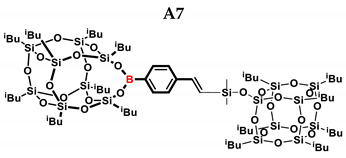


**^1^H NMR** (400 MHz, C_6_D_6_): δ (ppm) = 8.09–8.04 (m, 2H, B-C_6_H_4_) 7.49–7.40 (m, 2H, B-C_6_H_4_) 6.61–6.56 (d, J = 20.20 Hz, β isomer, B-C_6_H_4_-CH_=_CH-Si), 5.96–5.95 (d, J = 2.95 Hz, α isomer, B-C_6_H_4_-C(_=_CH_2_)-Si), 5.88–5.87 (d, J = 3.10 Hz, α isomer, B-C_6_H_4_-C(_=_CH_2_)-Si), 2.21–2.05 (m, -CH_2_-CH(CH_3_)_2_, 1.15–1.14 (m, -CH_2_-CH(CH_3_)_2_, 9 and 13 position isobutyl substituents), 1.09–1.06 (m, -CH_2_-CH(CH_3_)_2_), 0.85–0.78,(m -CH_2_-CH(CH_3_)_2_, 0.42–0.35 (m, SiMe_2_).

**^13^C NMR** (101 MHz, C_6_D_6_): δ (ppm) = 151.89, 146.82, 145.64, 141.09, 137.86, 136.10, 135.59, 131.68, 129.33, 128.56, 126.69, 126.47, 125.70, 125.57 (Ar), 26.24, 26.21, 26.14, 26.11, 26.10, 26.03, 26.00, 25.97, 24.50, 24.47, 24.43, 24.38, 23.53, 23.06, 23.02 (iBu), 0.49 (SiMe_2_).

**^29^Si NMR** (79.5 MHz, C_6_D_6_): δ (ppm) = 0.47, −0.17 (SiMe_2_), −66.60, −66.87, −67.54, −67.99, −68.83, −69.98, −70.04, −70.14, −70.41(cages), −109.13, −109.38 (SiO_4_).

**MALDI-TOF-MS:** [M] + Na^+^: 1913.684 (measured), 1913.624 (calculated).


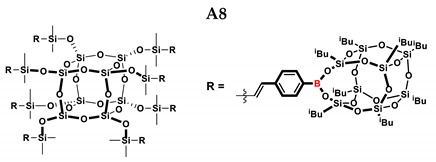


**^1^H NMR** (400 MHz, C_6_D_6_): δ (ppm) = 8.04–8.03 (m, 8H, B-C_6_H_4_) 7.40–7.36 (m, 8H, B-C_6_H_4_) 7.13–7.00 (m, 16H, B-C_6_H_4_) 6.55–6.49 (d, J = 19.38 Hz, β isomer, B-C_6_H_4_-CH_=_CH-Si), 6.00–5.95 (d, J = 19.23 Hz, β isomer, B-C_6_H_4_-CH_=_CH-Si), 2.21–2.05 (m, -CH_2_-CH(CH_3_)_2_, 1.17–1.16, -CH_2_-CH(CH_3_)_2_, 9 and 13 position isobutyl substituents), 1.10–1.06 (m, -CH_2_-CH(CH_3_)_2_), 0.94–0.93, (m, -CH_2_-CH(CH_3_)_2_, 9 and 13 position isobutyl substituents), 0.84–0.81 (m, -CH_2_-CH(CH_3_)_2_), 0.42–0.20 (m, SiMe_2_).

**^13^C NMR** (101 MHz, C_6_D_6_): δ (ppm) =151.39, 145.80, 141.09, 137.87, 136.09, 135.59, 131.69, 129.33, 128.56, 126.56, 125.70, 125.58 (Ar), 26.27, 26.13, 26.07, 26.04, 25.99, 25.96, 25.95, 24.73, 24.66, 24.52, 24.50, 24.49, 24.46, 24.39, 24.25, 23.54, 23.52, 23.49, 23.06, 23.03 (iBu), 1.02, 0.47 (SiMe_2_).

**^29^Si NMR** (79.5 MHz, C_6_D_6_): δ (ppm) = 1.95 (SiMe_2_), −66.85, −66.88, −67.99, −68.81, −70.07 (cage), −108.48 (SiO_4_).

**MALDI-TOF-MS:** The product mass exceeded the m/z ratio of the analytical instrument, and therefore MALDI-TOF-MS analysis was omitted.


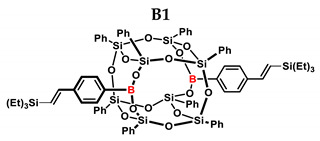


**^1^H NMR** (400 MHz, C_6_D_6_): δ (ppm) = 7.96–7.90 (m, 12H, Ph, B-C_6_H_4–_4-CH=CH-Si), 7.71–7.68 (m, 8H, Ph), 7.22–6.87 (m, 28H, Ph, B-C_6_H_4_-4-CH=CH, solvent), 6.92–6.87 (d, J = 19.29 Hz, β isomer, B-C_6_H_4_-CH_=_CH-Si), 6.44–6.39 (d, J = 19.29 Hz, β isomer, B-C_6_H_4_-CH_=_CH-Si), 5.80–5.79 (d, J = 3.10 Hz, α isomer, B-C_6_H_4_-C(_=_CH_2_)-Si), 5.48–5.47 (d, J = 3.08 Hz, α isomer, B-C_6_H_4_-CH_=_CH-Si), 1.00–0.86 (18H, 1.00–0.96,t, β isomer Si-CH_2_-CH3,0.90–0.86, t, α isomer Si-CH_2_-CH_3_), 0.65–0.52 (m, 12H, Si-CH_2_-CH_3_).

**^13^C NMR** (101 MHz, C_6_D_6_): δ (ppm) = 150.54, 148.94, 145.29, 141.39, 137.51, 136.39, 136.16, 134.37, 134.22, 134.19, 131.45, 130.73, 130.68, 130.60, 130.45, 130.42, 130.38, 128.95, 128.10, 127.92, 127.84, 127.68, 127.44, 126.10, 125.84, 125.32(Ph), 7.30 (CH_3_), 3.47 (CH_2_).

**^29^Si NMR** (79.5 MHz, C_6_D_6_): δ (ppm) = 2.28, −0.22 (SiEt_3_), −77.66, −79.52 (cage).

**MALDI-TOF-MS:** [M] + Na^+^: 1543.350 (measured), 1543.347 (calculated).


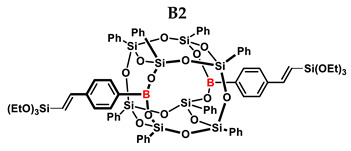


**^1^H NMR** (400 MHz, C_6_D_6_): δ (ppm) = 7.94–7.65 (m, 20H, Ph, B-C_6_H_4_-4-CH=CH-Si), 7.06–7.01 (m, 28H, Ph, B-C_6_H_4_-4-CH=CH, solvent), 6.66–6.14 (B-C_6_H_4_-CH_=_CH-Si), 4.08–3.86 (m, OCH_2_CH_3_), 1.23–0.90 (m, OCH_2_CH_3_).

**^13^C NMR** (101 MHz, C_6_D_6_): δ (ppm) = 136.21, 134.13, 131.49, 130.64, 130.55, 128.96, 127.68, 126.38, 125.32 (Ph), 59.94, 58.86, 58.42, 57.69 (CH_2_), 18.26, 18.16, 17.94, 17.30, 16.96 (CH_3_).

**^29^Si NMR** (79.5 MHz, C_6_D_6_): δ (ppm) = −77.61, −78.06, −79.03 (cage).


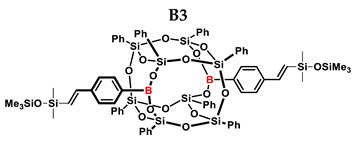


**^1^H NMR** (400 MHz, C_6_D_6_): δ (ppm) = 7.97–7.91 (m, 12H, Ph, B-C_6_H_4_-4-CH=CH-Si), 7.71–7.69 (m, 8H, Ph), 7.26–7.21 (m, 4H α isomer, Ph), 7.13–6.96 (m, 24H, Ph, B-C_6_H_4_-4-CH=CH, solvent), 6.47–6.42 (d, J = 19.12 Hz, β isomer, B-C_6_H_4_-CH_=_CH-Si), 5.82 (d, J = 2.37 Hz, α isomer, B-C_6_H_4_-C(_=_CH_2_)-Si), 5.64–5.63 (d, J = 2.33 Hz, α isomer, B-C_6_H_4_-CH_=_CH-Si), 0.23 (s, β isomer SiMe_2_), 0.20 (s, α isomer SiMe_2_), 0.15–0.14 (s, β isomer SiMe_3_), 0.05 (s, α isomer SiMe_3_).

**^13^C NMR** (101 MHz, C_6_D_6_): δ (ppm) = 153.32, 147.68, 145.17, 141.69, 138.12, 137.01, 136.80, 134.96, 134.84, 134.82, 134.74, 132.08, 131.36, 131.31, 131.24, 131.22, 131.09, 131.05, 131.01, 130.28, 129.58, 128.81, 128.72, 128.54, 128.47, 128.42, 128.30, 128.18, 128.06, 126.93, 126.67, 126.60, 125.94, (Ph), 2.48, 2.40, 2.35, 2.23, 1.38, 1.20 (SiMe_2_, SiMe_3_).

**^29^Si NMR** (79.5 MHz, C_6_D_6_): δ (ppm) = 8.38, 8.14 (OSiMe_3_), −3.15, −3.61 (OSiMe_2_), −77.70, −79.54 (cage).

**MALDI-TOF-MS:** [M] + Na^+^: 1607.230 (measured), 1607.291 (calculated).


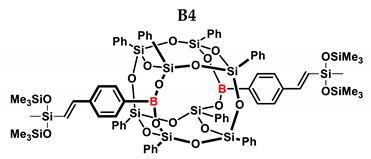


**^1^H NMR** (400 MHz, C_6_D_6_): δ (ppm) = 7.97–7.90 (m, 12H, Ph, B-C_6_H_4_-4-CH=CH-Si), 7.70–7.69 (m, 8H, Ph), 7.37–7.21(m, 4H α isomer, Ph), 7.16–6.96 (m, 24H, Ph, B-C_6_H_4_-4-CH=CH, solvent), 6.42–6.38 (d, J = 19.20 Hz, β isomer, B-C_6_H_4_-CH_=_CH-Si), 5.88 (d, J = 2.94 Hz, α isomer, B-C_6_H_4_-C(_=_CH_2_)-Si), 5.76–5.75 (d, J = 2.92 Hz, α isomer, B-C_6_H_4_-CH_=_CH-Si), 0.27 (s, β isomer SiMe), 0.24 (s, α isomer SiMe), 0.18 (s, β isomer SiMe_3_), 0.08 (s, α isomer SiMe_3_).

**^13^C NMR** (101 MHz, C_6_D_6_): δ (ppm) = 151.20, 146.66, 146.64, 145.82, 141.35, 136.81, 136.54, 134.75, 134.60, 134.58, 134.56, 131.86, 131.83, 131.81, 131.78, 131.13, 131.11, 131.08, 131.05, 131.02, 130.98, 130.94, 130.85, 130.80, 130.75, 129.33, 128.74, 128.57, 128.49, 128.47, 128.45, 128.30, 128.22, 128.20, 128.17, 128.06, 127.94, 127.82, 126.85, 126.38, 125.70 (Ph), 2.08, 1.61, 0.31(SiMe, SiMe_3_).

**^29^Si NMR** (79.5 MHz, C_6_D_6_): δ (ppm) = 8.23, 8.04 (OSiMe_3_), −33.38, −36.05 (OSiMe), −77.64, −77.71, −79.55, −79.56 (cage).

**MALDI-TOF-MS:** [M] + Na^+^: 1755.336 (measured), 1755.329(calculated).


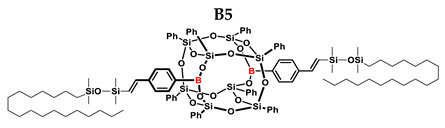


**^1^H NMR** (400 MHz, C_6_D_6_): δ (ppm) = 7.99–7.77 (m, 12H, Ph, B-C_6_H_4_-4-CH=CH-Si), 7.71–7.67 (m, 8H, Ph), 7.26–7.22(m, 4H α isomer, Ph), 7.13–6.97 (m, 24H, Ph, B-C_6_H_4_-4-CH=CH, solvent), 6.49–6.44 (d, J = 19.18 Hz, β isomer, B-C_6_H_4_-CH_=_CH-Si), 5.84–5.83 (d, J = 2.68 Hz, α isomer, B-C_6_H_4_-C(_=_CH_2_)-Si), 5.67 (d, J = 2.72 Hz, α isomer, B-C_6_H_4_-CH_=_CH-Si), 1.53–1.20 (m, 64H, octadecyl chain -CH_2_-), 0.96–0.88 (m, 6H. octadecyl chain -CH_3_), 0.68–0.52 (m, 4H, octadecyl SiCH_2_-, SiCH-), 0.26–0.01 (s, 24H, SiMe_2_).

**^13^C NMR** (101 MHz, C_6_D_6_): δ (ppm) = 153.17, 147.48, 144.93, 141.47, 137.87, 136.79, 136.58, 136.15, 134.60, 134.58, 134.50, 131.87, 131.84, 131.82, 131.61, 131.10, 131.04, 131.00, 130.97, 130.86, 130.82, 130.78, 130.75, 130.72, 130.07, 129.34, 128.70, 128.57, 128.46, 128.30, 128.22, 128.17, 128.06, 127.94, 127.82, 126.69, 126.35, 125.70 (Ph), 34.02, 33.97, 33.92, 32.39, 30.30, 30.27, 30.23, 30.17, 30.14, 30.12, 29.99, 29.93, 29.87, 26.00, 23.91, 23.86, 23.76, 23.17, 21.48, 18.92, 18.86, 18.75, 14.43 (CH_2_, CH_3_), 1.25, 1.05, 0.76, 0.72, 0.69, 0.55, 0.50(SiMe_2_).

**^29^Si NMR** (79.5 MHz, C_6_D_6_): δ (ppm) = 8.80, 8.54, −3.36, −3.83 (SiMe_2_), −77.72, −79.48, −79.59 (cage).

**MALDI-TOF-MS:** [M] + Na^+^: 2083.830 (measured), 2083.823 (calculated).


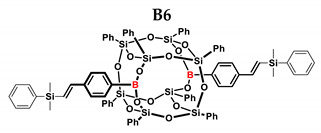


**^1^H NMR** (400 MHz, C_6_D_6_): δ (ppm) = 7.93–7.85 (m, 12H, Ph, B-C_6_H_4_-4-CH=CH-Si), 7.71–7.68 (m, 8H, Ph), 7.51–7.45, 7.23–6.93 (m, 38H, Ph, B-C_6_H_4_-4-CH=CH, solvent), 6.55–6.50 (d, J = 19.06 Hz, β isomer, B-C_6_H_4_-CH_=_CH-Si), 5.87–5.86 (d, J = 2.27 Hz, α isomer, B-C_6_H_4_-C(_=_CH_2_)-Si), 5.56–5.55 (d, J = 2.37 Hz, α isomer, B-C_6_H_4_-CH_=_CH-Si), 0.34–0.19 (m, 24H SiMe_2_).

**^13^C NMR** (101 MHz, C_6_D_6_): δ (ppm) = 151.59, 148.04, 145.88, 141.47, 137.89, 136.73, 136.50, 136.33, 134.77, 134.39, 134.34, 134.31, 133.93, 131.82, 131.79, 131.12, 130.99, 130.79, 130.75, 129.67, 129.38, 129.34, 128.74, 128.57, 128.48, 128.44, 128.30, 128.23, 128.19, 128.06, 127.94, 127.82, 126.69, 126.52, 126.38, 125.70 (Ph), 1.45, 0.52, −0.22, −2.19, −2.44, −3.04 (SiMe_2_).

**^29^Si NMR** (79.5 MHz, C_6_D_6_): δ (ppm) = 8.45, 10.63 (SiMe_2_), −77.68, −79.54 (cage).

**MALDI-TOF-MS:** [M] + Na^+^: 1583,290 (measured), 1583.285 (calculated).


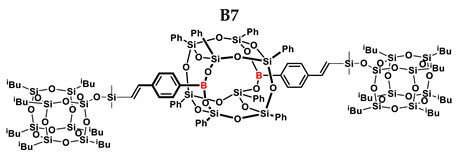


**^1^H NMR** (400 MHz, C_6_D_6_): δ (ppm) = 7.95–7.81 (m, 12H, Ph, B-C_6_H_4_-4-CH=CH-Si), 7.69–7.67 (m, 8H, Ph), 7.27–7.22(m, 4H α isomer, Ph), 7.13–6.97 (m, 24H, Ph, B-C_6_H_4_-4-CH=CH, solvent), 6.51–6.46 (d, J = 19.20 Hz, β isomer, B-C_6_H_4_-CH_=_CH-Si), 6.40–6.36 (d, J = 19.23 Hz, β isomer, B-C_6_H_4_-CH_=_CH-Si), 5.95 (d, J = 2.37 Hz, α isomer, B-C_6_H_4_-C(_=_CH_2_)-Si), 5.82–5.81 (d, J = 2.33 Hz, α isomer, B-C_6_H_4_-CH_=_CH-Si), 2.09–1.99 (m, 14H, -CH_2_-CH(CH_3_)_2_), 1.08–1.02 (m, 84H, -CH_2_-CH(CH_3_)_2_), 0.83–0.77 (m, 28H, -CH_2_-CH(CH_3_)_2_), 0.42 (s, α isomer, SiMe_2_), 0.35–0.33 (d, β isomer SiMe_2_).

**^13^C NMR** (101 MHz, C_6_D_6_): δ (ppm) = 151.80, 147.07, 145.58, 141.30, 137.87, 136.71, 136.64, 136.14, 134.59, 134.58, 131.86, 131.04, 130.77, 129.33, 128.57, 128.45, 128.21, 126.66, 126.45, 125.70 (Ph,Ar), 25.98, 25.95, 24.43, 24.41, 24.36, 24.34, 23.02, 23.00, 22.93, 21.47 (iBu), 0.54, 0.47 (SiMe_2_).

**^29^Si NMR** (79.5 MHz, C_6_D_6_): δ (ppm) = 0.45, −0.26 (SiMe_2_), −66.58, −67.51, −67.53, −67.55, −77.69, −77.72, −79.62, −79.65 (cages), −109.14, −109.38 (SiO_4_).

**MALDI-TOF-MS:** The product mass exceeded the m/z ratio of the analytical instrument, and therefore the MALDI-TOF-MS analysis was omitted.

### 3.4. Analytical Methods

The ^1^H NMR spectra were recorded on a Bruker Ultrashield 300 MHz (Bruker, Poznań, Poland). The ^13^C and ^29^Si NMR spectra were recorded on a Bruker Ascend 400 MHz (Bruker, Poznań, Poland) operating at 101 and 79 MHz, respectively. Benzene-d_6_ and CDCl_3_ was used as a solvent.

MALDI-TOF mass spectra were recorded on an UltrafleXtreme mass spectrometer (Bruker Daltonics), equipped with a SmartBeam II laser (355 nm) in the 500–4000 *m*/*z* range, and 2,5-dihydroxybenzoic acid (DHB, Bruker Daltonics, Bremen, Germany) served as the matrix.

Thermogravimetry (TG) was performed using a NETZSCH 209 F1 Libra gravimetric analyzer (Selb, Germany). Samples of 2 ± 0.2 mg were placed in Al_2_O_3_ crucibles. Measurements were conducted under nitrogen (flow of 20 mL/min) in the range of 50–1000 °C with a 20 °C/min heating rate.

A digital light microscope Keyence VHX 7000 (Keyence International, Mechelen, Belgium) with a 100 × 1000 VH-Z100T lens was used to examine the samples. All images were recorded with a VHX 7020 camera.

The Raman studies were carried out using a WITec Alpha 300M+ spectrometer (Ulm, Germany). A 488 nm laser with 600 gratings was chosen along with a 100× ZEISS objective (Oberkochen, Germany). Each sample was measured 3 times for 2 min each.

An analysis of the functional groups of the compounds was performed by ATR-FTIR analysis, and the spectra were scanned in the range of 4000–400 cm^−1^. A total of 16 scans were collected with 2 cm^−1^ resolution. The obtained spectra were subjected to baseline correction and normalization using Bruker OPUS 7.2 software (Billerica, MA, USA).

The melting point was measured using a Büchi M-565 analyzer (Flawil, Switzerland). Based on the optical parameters of the phase change course of the sample in the capillary, an automatic measurement was performed with the determination of the melting point (temperature increase of 5 °C/min). The result is the average of the three measurements.

## 4. Conclusions

In the described work, tests of the reactivity of new borasilsesquioxanes were carried out. A range of organosilicon compounds were obtained through catalytic hydrosilylation. During the tests, the mono- and diethynylphenylborasilsesquioxane derivatives showed higher reactivity than the vinyl counterpart. This resulted in a greater conversion rate when stoichiometric reagent amounts were used. The newly synthesized products were fully characterized using spectroscopic methods such as MALDI-TOF-MS and NMR. The compounds that have been derived can be used as building blocks and synthons for further organo-metallic synthesis. They can also be applied as doping agents for semi-conducting materials, as well as for silicon and boron-containing preceramic additives.

To the best of our knowledge, for the first time, an attempt was here made to characterize the process of thermal decomposition of borasilsesquioxanes. The individual stages of decomposition were thoroughly examined, and spectroscopic techniques such as NMR, ATR-FTIR, and Raman were used to confirm the structural changes that occurred. Additionally, the formation of polymeric structures during the heating of diethynylphenylborasilsesquioxane was determined through this study.

In conclusion, the distinct compositions and characteristics of silsesquioxanes continue to be an intriguing subject of study, with significant potential for utilization in novel materials and technologies. Further investigation into their capabilities is likely to uncover even more valuable applications for these innovative compounds.

## Figures and Tables

**Table 1:** Selectivity and conversion of monoethynylphenylborasilsesquioxane with Si–H-bearing substrates.

Entry	Silane Compound	Molar Ratio ^[a]^	Conversion/Yield ^[b]^	α:β Ratio ^[b]^
A1	Et_3_SiH	1:1	99%	34:66
A2	(EtO)_3_SiH	1:1	~65%	~62:38
A3	PMDS	1:1	84%	43:57
		1:1.5	99%	
A4	HMTS	1:1	99%	52:48
A5	Me_2_PhSiH	1:1	99%	29:71
A6	TMDS-OD	1:1	80%	47:53
		1:1.5	99%	
A7	SSQ-OSiH	1:1	65%	55:45
A8	SS-8H	8:1	77%	4:96

**Conditions**: toluene, 110 °C, 10^−5^ Pt (per mol Si-H), 24 h, closed system. ^[a]^ Silsesquioxane to silane reagent molar ratio. ^[b]^ Calculated from ^1^H NMR on the basis of proton signal integration of −C≡C**H** moiety. PMDS: Pentamethyldisiloxane. HMTS: 1,1,1,3,5,5,5-heptamethyltrisiloxane. TMDS-OD: 1,1,3,3,-tetramethyloctadecylsiloxane. iBu_7_SSQ-OSiH: Dimethylsiloxyheptaisobutyloctasilsesquioxane. SS-8H: Octaspherosilicate.

**Table 2:** Selectivity and conversion of diethynylphenylborasilsesquioxane with Si–H-bearing substrates.

Entry	Silane Compound	Molar Ratio ^[a]^	Conversion/Yield ^[b]^	α:β Ratio ^[b]^
B1	Et_3_SiH	1:1	97%	29:71
B2	(EtO)_3_SiH	1:1	~99%	-
B3	PMDS	1:1	98%	40:60
B4	HMTS	1:1	99%	37:63
B5	Me_2_PhSiH	1:1	99%	25:75
B6	TMDS-OD	1:1	90%	44:56
		1:1.5	99%	
B7	SSQ-OSiH	1:1	92%	31:69

**Conditions**: toluene, 110 °C, 10^−5^ Pt (per mol Si-H), 24 h, closed system. ^[a]^ Silsesquioxane to silane reagent molar ratio. ^[b]^ Calculated from ^1^H NMR on the basis of proton signal integration of −C≡C**H** moiety.

**Table 3:** Results of thermogravimetric analysis.

Sample	1% Mass Loss (°C) T_1%_	Onset Temperature (°C) T_onset_				Temperature at the Maximum Rate of Mass Loss (°C) T_max_			
	-	I	II	III	IV	I	II	III	IV
A	216.2	224.2	330.0	422.6	512.4	266.8	365.8	429	521.8
B	283.1	278.1	389.0	504.4	560.5	284.4	441.5	602.1	-

## Data Availability

Not applicable.
